# Microvessel Density in Patients with Cutaneous Melanoma: An Up-to-Date Systematic Review and Meta-Analysis

**DOI:** 10.1155/2017/2049140

**Published:** 2017-12-26

**Authors:** Konstantinos Perivoliotis, Panagiotis Ntellas, Katerina Dadouli, Prodromos Koutoukoglou, Maria Ioannou, Konstantinos Tepetes

**Affiliations:** ^1^Department of Surgery, University Hospital of Larissa, Mezourlo, 41110 Larissa, Greece; ^2^Department of Pathology, University Hospital of Larissa, Mezourlo, 41110 Larissa, Greece; ^3^Postgraduate Programme (MSc), Research Methodology in Biomedicine, Biostatistics and Clinical Bioinformatics, University of Thessaly, Larissa, Greece

## Abstract

**Background:**

We conducted a meta-analysis, in order to appraise the effect of microvessel density (MVD) on the survival of patients with cutaneous melanoma.

**Methods:**

This study was conducted according to the PRISMA guidelines and the Cochrane Handbook for Systematic Reviews of Interventions. A systematic literature search in electronic databases (MEDLINE, Web of Science, and Cochrane Central Register of Controlled Clinical Trials) was performed. Fixed Effects or Random Effects model was used, based on the Cochran *Q* test.

**Results:**

In total 9 studies (903 patients) were included. Pooled HR for overall survival (OS) and disease-free survival (DFS) were 2.62 (95% CI: 0.71–9.60, *p* = 0.15) and 2.64 (95% CI: 0.82–8.47, *p* = 0.10), respectively. Odds ratios of overall survival between high and low MVD groups, at 12 (1.45, 95% CI: 0.16–13.24), 36 (2.93, 95% CI: 0.63–13.59), and 60 (4.09, 95% CI: 0.85–19.77) months did not reach statistical significance. Significant superiority of low MVD group, in terms of DFS, at all time intervals (OR: 4.69, *p* < 0.0001; OR: 2.18, *p* = 0.004; OR: 7.46, *p* = 0.01, resp.) was documented.

**Discussion:**

MVD does not affect the HR of OS and DFS. A strong correlation with DFS rates at 12, 36, and 60 months was recorded.

## 1. Introduction

### 1.1. Rationale

Melanoma is defined as the malignancy deriving from pigment containing cells, also known as melanocytes, and is located mainly in the skin. Cutaneous melanoma is not a single neoplastic disease, since it consists of several subtypes, such as superficial spreading melanoma, lentigo maligna, acral lentiginous melanoma, and nodular melanoma.

Although cutaneous melanoma represents a small proportion of all skin cancers, it is directly associated with the majority of skin cancer-related deaths [1]. Moreover, the overall incidence of cutaneous melanoma has an increasing trend over the past decades, with variations being reported between different geographic areas, populations, and genders [2–5].

Several studies have attempted to identify prognostic factors for the overall survival. Among the proposed indicators were both clinical parameters and tumor characteristics, such as age, sex, Breslow index, ulceration, anatomic site, Clark level, mitotic rate, histological regression, and vascularity [6, 7].

Angiogenesis, the formation of novel blood vessels, is a naturally occurring procedure and is observed in processes like embryonic growth and wound healing. Angiogenesis has, also, been recognized as a key determinant factor in cancer growth and metastases development in hematologic malignancies [8, 9] and solid tumors, such as breast, gastric, colorectal, and pancreatic tumors [10]. Tumor vascularization is located in the dermis and in thin melanomas and is associated with the vertical growth phase. According to the literature, extensive angiogenesis in cutaneous melanomas displayed a 69% risk of relapse and a 42% mortality rate, when compared to a 33% and 12% respective rate of vascularity absent tumors [7].

Microvessel density (MVD) counting, as described by Weidner et al. [11], allows, through the application of immunohistochemical stains, like von Willebrand factor (vWF), cluster of differentiation (CD) 31, CD34, and CD105, the quantification of the vasculature of the tumors.

A respectable amount of studies has investigated the prognostic value of MVD in cutaneous melanoma, with inconclusive results. More specifically, although many reports directly correlate tumor MVD and survival rates [12, 13], Hillen et al. [14] found that microvessel density is not associated with tumor stage or survival. Furthermore, a meta-analysis by Pastushenko et al. [15] concluded that MVD does not have a prognostic value for melanoma.

### 1.2. Objectives

In light of this conflicting evidence, we conducted a systematic literature review and a meta-analysis, in order to provide an up-to-date insight of the current literature and appraise the effect of intratumoral vascularity, through MVD measurements, on the survival of patients with cutaneous melanoma.

## 2. Methods

### 2.1. Study Protocol

The present study was conducted according to the PRISMA guidelines [16] and the Cochrane Handbook for Systematic Reviews of Interventions. This meta-analysis was not registered in any electronic database.

### 2.2. Primary Endpoint

Primary endpoint of this meta-analysis was the pooled Hazard Ratio (HR) for the overall survival (OS), between high and low MVD measurements, in patients with cutaneous melanoma. Pooled HR > 1 indicated higher risk of death in patients with high MVD, against patients with low MVD.

### 2.3. Secondary Endpoints

Secondary endpoints included the pooled Odds Ratios (ORs) of overall survival and disease-free survival (DFS), between high and low MVD measurements, in three fixed time points. More specifically, pooled ORs were calculated for the first year (12 months), the third year (36 months), and the fifth year (60 months) of follow-up. Pooled OR > 1 indicated superiority, in terms of survival, of the patients with low MVD against the patients with high MVD.

### 2.4. Eligibility Criteria

Eligibility criteria for this meta-analysis were (1) trials with a study population consisting of patients with cutaneous melanoma, (2) primary tumor MVD assessment, (3) reporting outcomes of interest, (4) retrievable study results, and (5) article written in English.

Excluded studies included those not written in English, with no outcome of interest, and with insufficient data and nonhuman studies. Furthermore, trials in the form of letters, conference abstracts, expert opinion, or duplicate studies were excluded.

### 2.5. Literature Search

In order to identify eligible studies, a systematic literature search in electronic databases (MEDLINE, Web of Science, and Cochrane Central Register of Controlled Clinical Trials) was performed. The last search date was June 2017.

The following search algorithm was used:

- MELANOMA AND (MVD OR MICROVASCULAR DENSITY OR MICROVESSEL DENSITY).

### 2.6. Study Selection and Data Collection

The first step of the literature screening included removal of the duplicate studies. After the removal of the duplicate entries, the titles and the abstracts of the studies were screened on the basis of the eligibility criteria. The next step included a full text review of the remaining articles, in order to assess consistency with the inclusion criteria. Electronic database screening, study selection, data extraction, and methodological and quality rating were performed in duplicate and blindly by two independent researchers (D. K. and K. P.). In case of a discrepancy, through mutual revision and discussion, a consensus was reached. If disagreements were not resolved, the opinion of a third researcher was considered (P. K.)

The extracted data from the eligible trials included first author's name, study type, trial location and year, sample size, age and gender of the patients, duration of follow-up, MVD assessment method, cut-off value for MVD, categorization of tumor based on Clark's level and Breslow thickness, location and histotype of melanoma, and information regarding the treatment applied. Furthermore, data involving overall HR and OR of overall and disease-free survival at the specified three time points (12, 36, and 60 months) were, also, retrieved. In case of data from both peritumoral and intratumoral areas, only results of samples from the malignancy core were used [17, 18]. Extraction was performed only for results reported in the article of the studies.

All studies incorporated in the meta-analysis underwent rigorous quality and methodological evaluation according to the Newcastle-Ottawa Scale (NOS) [19]. The above-mentioned assessment tool evaluates non-RCT reports in certain validity checkpoints such as the selection and the comparability of the study groups and the confirmation of the exposure. Every study was appointed a score of 0–9. Cohen's *k* statistic was also calculated.

### 2.7. Statistical Analysis

The Cochrane Collaboration RevMan version 5.3 was used for the completion of data analysis. Primary and secondary endpoints were reported in the form of HR and OR, respectively. All analyses' results were apposed with the corresponding 95% Confidence Interval (95% CI).

It must be noted that, in case that HR and OR were not directly provided in the article results, they were estimated according to the methods described by Parmar et al. [20] and Tierney et al. [21]. More specifically, from the published Kaplan-Meier (KM) curves, the necessary data for the estimation of the HR and the ORs were reconstructed [22]. In order to achieve maximum precision in the data extraction from the KM curves, a digitizing software (Digitizelt) was used [23].

If the study report did not provide the mean and the Standard Deviation (SD) of continuous variables, they were calculated from the median and the Interquartile Range (IR), based on the formula by Hozo et al. [24]. More specifically, for a sample size > 25, the mean was considered equal to the median. If the sample size was <70, then, SD was equal to IR/4. Furthermore, for a sample size > 70, SD derived from the formula IR/6.

The statistical method applied was the Mantel-Haenszel (MH) and the Inverse Variance (IV), for OR and HR, respectively. Both Fixed Effects (FE) and Random Effects (RE) models were calculated. The model that was finally estimated was based on the Cochran *Q* test. In case of a statistically significant heterogeneity (*Q* test *p* < 0.1), the RE model was applied. Otherwise, the pooled results estimation was based on the FE model. Overall heterogeneity was measured in terms of *I*^2^. Statistical significance was considered at the level of *p* < 0.05.

### 2.8. Risk of Bias Across Studies

The possible presence of publication bias was determined, primarily, by visual inspection of the funnel plot of the primary outcome. Moreover, as far as the primary outcome was considered, an Egger's test was also performed.

## 3. Results

### 3.1. Study Selection

**Figure 1:**
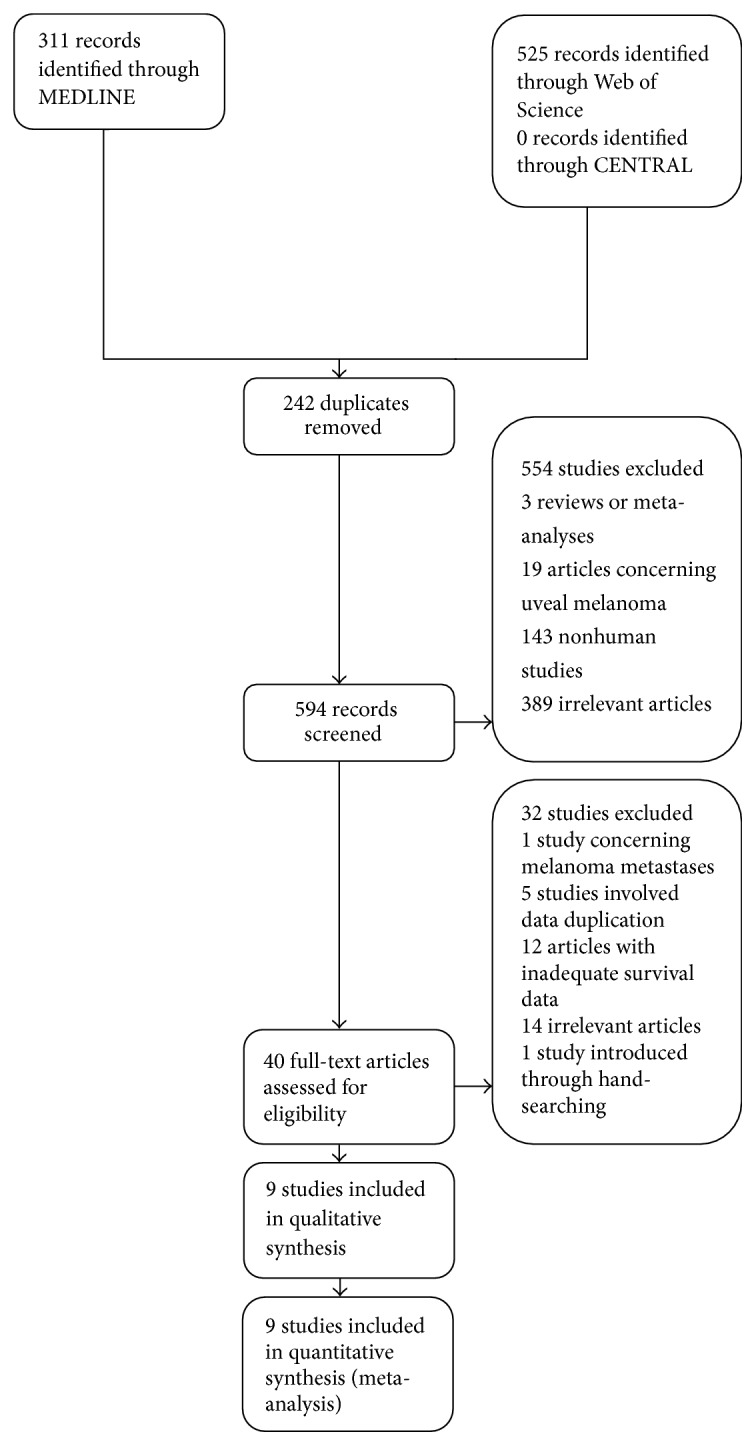
Flow diagram.

Electronic database search resulted in the retrieval of 836 entries (Figure 1). More specifically, 311 and 525 articles were identified from MEDLINE and Web of Science, respectively. No trial was found from CENTRAL database. After the duplicate removal, 594 records were submitted to the first step of the screening. Review of titles and abstracts resulted in the exclusion of 554 studies. From the above-mentioned articles, 3 concerned reviews or meta-analyses, 19 focused on uveal melanoma, 143 were animal studies, and 389 were irrelevant to the subject records. Full text assessment, according to the eligibility criteria, was performed in 40 articles, resulting in the removal of 32 studies. During this phase, 1 study [12] analyzed samples from melanoma metastases, 5 studies [25–29] featured data duplication, 12 articles [18, 30–40] did not report adequate, for the meta-analysis, survival data, and 14 entries did not consider a relevant subject. Furthermore, 1 study [41] was introduced through hand-searching of the existing literature. Finally, 9 studies [13, 14, 17, 41–46] were included in the qualitative and quantitative analysis.

### 3.2. Study Characteristics

The characteristics of the included studies are summarized in Table 1. Regarding the study type, only two studies [17, 43] were conducted in a prospective manner, while the rest of the included trials had a retrospective design. The publication date spanned from 1999 to 2015. Except the study of Pastushenko et al. [46] which was multicentered, all other trials were performed in a single center. In total, the included sample was 903 patients, while the total amount of the sample that provided survival data was 875. Moreover, 1008 specimens were excised and analyzed. Despite the fact that two studies [17, 42] did not provide adequate data, the age of the included patients ranged from 18 to 90 years. The gender allocation of the subjects of each included trial is apposed in Table 1. As far as the duration of follow-up was concerned, although in most trials the mean follow-up value fluctuated around the value of 5 years, in some cases [14] it extended up to 10 years.

Regarding the method that was applied for the MVD assessment, the majority of the eligible articles reported the use of light microscopy and immunochemistry, based on the technique first described by Weidner et al. [11] (Table 2). Transmission electron microscopy [17] and the Chalkley score [46] were also used in some studies. The most frequently utilized antibody for the evaluation of MVD was CD-31. However, VIII factor antibodies were applied by the study groups of Straume and Akslen [42] and Ribatti et al. [43]. Furthermore, references for the use of anti-laminin [17] and CD-34 [14, 44, 46] antibodies were recorded. Heterogeneity was observed in the magnification utilized, which ranged from 10x to 400x, and in the number of spots examined. Blinded reading by at least two observers was recorded in four studies [13, 14, 43, 44]. It must, also, be noted that separate counting for intra- and peritumoral vessels was performed in only four of the included trials [14, 17, 42, 46]. Moreover, as described in Table 2, lack of homogeneity was identified in the MVD cut-off level.

Table 3 summarizes the location characteristics of the malignancies under study. More specifically, the localization of the tumors was 114 in the area of head and neck, 215 in the trunk, 373 in the extremities, and 75 in the genital area. A total of 282 cases reported signs of melanoma ulceration. Totally, four studies [13, 41, 42, 45] provided categorization of the melanomas based on Clark's classification system, while heterogeneity existed between the studies, in the definition of the tumor thickness subgroups (Table 4). Information concerning the tumor histotype was scarcely quoted in the included articles [14, 45] (Table 5). Similarly, despite the fact that surgical excision of the primary tumor was extensively applied, data regarding the administration of adjuvant chemotherapy were not systematically reported.

### 3.3. Risk of Bias within Studies

Methodological and quality rating of the included trials is quoted in Table 6. Consistent results were yielded regarding the overall study score, with eight studies being awarded with 5 stars and one study [43] 6 stars. The strength of interrater agreement was estimated to be in a more than adequate level (Cohen's *k* statistic: 91.3%, *p* < 0.001).

### 3.4. Primary Endpoint

**Figure 2:**
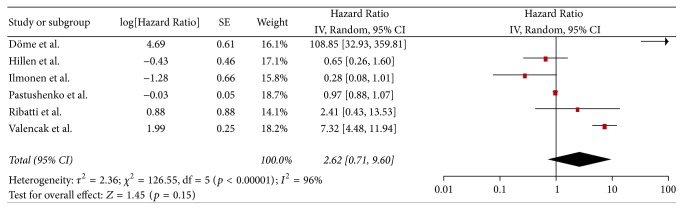
Hazard Ratio of overall survival.

- Data regarding the Hazard Ratio of OS were extracted from 6 studies [13, 14, 17, 41, 43, 46] (Figure 2). Meta-analysis of these data showed no statistically significant (*p* = 0.15) Hazard Ratio for OS between high and low MVD groups (HR: 2.62, 95% CI: 0.71–9.60). As a result of the significant heterogeneity between the studies (*Q* test *p* < 0.00001, *I*^2^ = 96%), a RE model was applied.
- Due to the high level of heterogeneity that was outlined previously, further analysis was performed. Sensitivity analysis for each study separately did not affect the level of heterogeneity, which remained statistically significant (*Q* test *p* < 0.00001, *I*^2^: 94–97%). Subgroup analysis, on the basis of the antibody used, did not highlight any statistically significant difference regarding the pooled HR, while decreased heterogeneity was noted, in the CD34 arm (HR: 0.97, 95% CI: 0.88–1.06, *Q* test *p* = 0.39, *I*^2^ = 0%), but not in the respective CD31 group (HR: 3.49, 95% CI: 0.42–29.03, *Q* test *p* < 0.0001, *I*^2^ = 95%). Metaregression for the variables of age and MVD cut-off did not yield any statistically significant results (*p* = 0.768 and *p* = 0.287, resp.). Analysis in terms of Clark's level, Breslow thickness, location, and melanoma histotype was not performed due to scarcity or inconsistency of the reported data.

### 3.5. Secondary Endpoints

- In total, 5 studies [13, 14, 17, 41, 43] provided data concerning the comparison between high and low MVD for OS at 12 months (Figure 4). Meta-analysis of these data showed no statistically significant (*p* = 0.74) difference of OS (OR: 1.45, 95% CI: 0.16–13.24) at 12 months between the two groups. Heterogeneity was significant between the studies (*Q* test *p* = 0.07, *I*^2^ = 57%) and therefore a RE model was applied.
- In total, 5 studies [13, 14, 17, 41, 43] provided data concerning the comparison between high and low MVD for OS at 36 months (Figure 5). Meta-analysis of these data showed no statistically significant (*p* = 0.17) difference of OS (OR: 2.93, 95% CI: 0.63–13.59) at 36 months between the two groups. Heterogeneity was significant between the studies (*Q* test *p* = 0.004, *I*^2^ = 74%) and therefore a RE model was applied.
- In total, 6 studies [13, 14, 17, 41, 43, 44] provided data concerning the comparison between high and low MVD for OS at 60 months (Figure 6). Meta-analysis of these data showed no statistically significant (*p* = 0.08) difference of OS (OR: 4.09, 95% CI: 0.85–19.77) at 60 months between the two groups. Heterogeneity was significant between the studies (*Q* test *p* < 0.00001, *I*^2^ = 87%) and therefore a RE model was applied.
- In total, 4 studies [13, 42, 45, 46] provided data concerning the Hazard Ratio of DFS (Figure 7). Meta-analysis of these data showed no statistically significant (*p* = 0.10) Hazard Ratio for DFS (HR: 2.64, 95% CI: 0.82–8.47) between the two groups. Heterogeneity was significant between the studies (*Q* test *p* < 0.00001, *I*^2^ = 97%) and therefore a RE model was applied.
- In total, 3 studies [13, 42, 45] provided data concerning the comparison between high and low MVD for DFS at 12 months (Figure 8). Meta-analysis of these data showed a statistically significant (*p* < 0.0001) higher ratio of DFS (OR: 4.69, 95% CI: 2.16–10.19) at 12 months in favor of the low MVD group. Heterogeneity was not significant between the studies (*Q* test *p* = 0.70, *I*^2^ = 0%) and therefore a FE model was applied.
- In total, 3 studies [13, 42, 45] provided data concerning the comparison between high and low MVD for DFS at 36 months (Figure 9). Meta-analysis of these data showed a statistically significant (*p* = 0.004) higher ratio of DFS (OR: 2.18, 95% CI: 1.28–3.70) at 36 months in favor of the low MVD group. Heterogeneity was not significant between the studies (*Q* test *p* = 0.69, *I*^2^ = 0%) and therefore a FE model was applied.
- In total, 4 studies [13, 42, 44, 45] provided data concerning the comparison between high and low MVD for DFS at 60 months (Figure 10). Meta-analysis of these data showed a statistically significant (*p* = 0.01) higher ratio of DFS (OR: 7.46, 95% CI: 1.55–35.97) at 60 months in favor of the low MVD group. Heterogeneity was significant between the studies (*Q* test *p* < 0.0001, *I*^2^ = 88%) and therefore a RE model was applied.

### 3.6. Risk of Bias Across Studies

**Figure 3:**
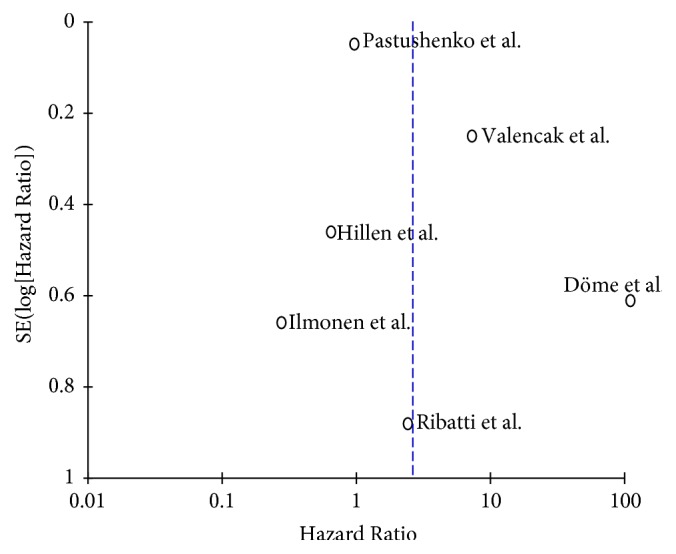
Funnel plot of primary endpoint.

**Figure 4:**
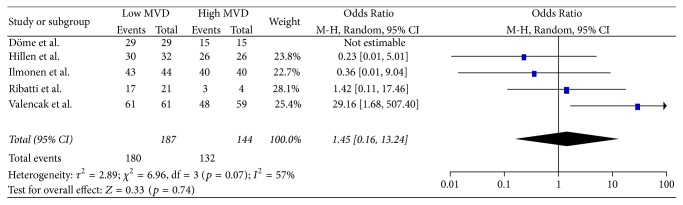
12 months' overall survival.

**Figure 5:**
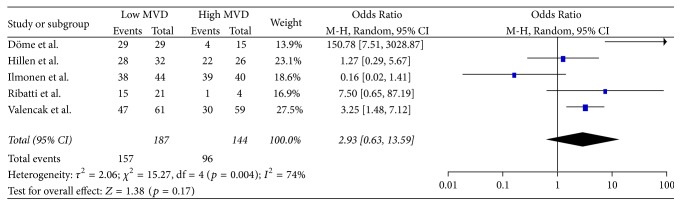
36 months' overall survival.

**Figure 6:**
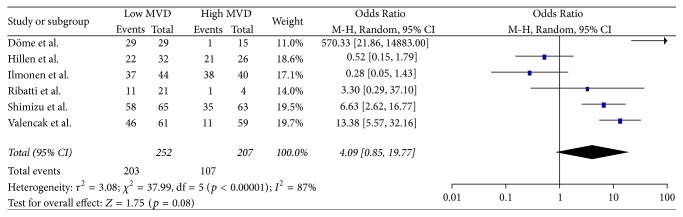
60 months' overall survival.

**Figure 7:**
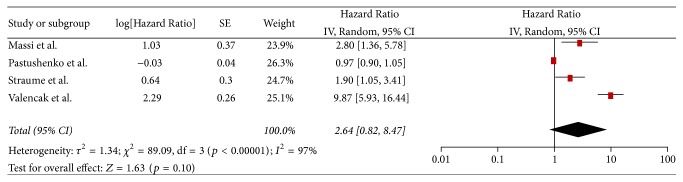
Hazard Ratio of disease-free survival.

**Figure 8:**
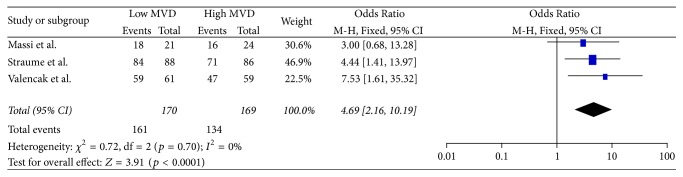
12 months' disease-free survival.

**Figure 9:**
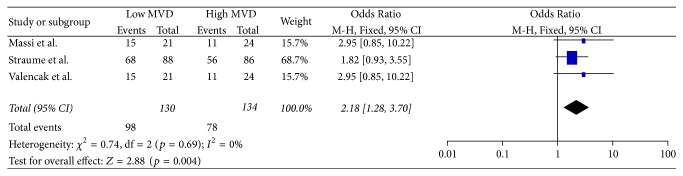
36 months' disease-free survival.

**Figure 10:**
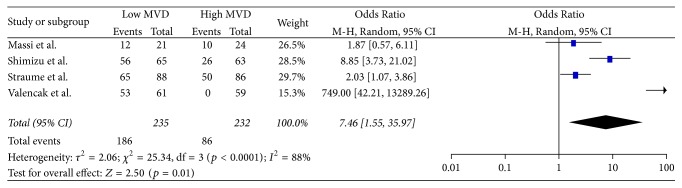
60 months' disease-free survival.

The funnel plot of the primary endpoint is apposed in Figure 3. Visual inspection of the graphical representation revealed a symmetrical distribution on both sides of the combined effect size line. According to Egger's test, there was no statistically significant publication bias (*p* = 0.305).

## 4. Discussion

### 4.1. Summary of Evidence

According to the current literature, after the steady increase during the 1960–1990 period, the rate of overall skin cancer tended to consolidate over specific levels [5]. Despite that, cutaneous melanoma illustrates a continuing increase in incidence, with significant variations, however, in the reported ratios. Several etiologic factors for this increase have been proposed, such as the increase of exposure of fair skin individuals to ultraviolet radiation and the augmentation of flights from high to low attitude countries. According to MacKie et al. [3], melanoma annual incidence rates ranged from 55.8/10^5^ for males and 41.1/10^5^ for females in Queensland, Australia, to 3.8/10^5^ for males and 4.8/10^5^ for females in Serbia. Forsea et al., however, in a recent study, claimed that reports from population-based cancer registries can be misleading due to lack of quality cancer registration in many countries and concluded that mortality-to-incidence ratio of cutaneous melanoma in Europe ranged from 0.09 to 0.44 [4].

Due to the high prevalence and mortality ratios of cutaneous melanoma, various prognostic factors have been investigated in the literature, including age, sex, tumor location, lymph node involvement, tumor thickness, ulceration, Clark level, tumor vascularity, lymphovascular invasion, microsatellites, mitotic rate, regression, tumor infiltrating lymphocytes, BRAF mutations, distant metastasis, and LDH [7]. Ulceration and tumor thickness have been widely recognized as, independently, having a major impact on survival [7]. More specifically, the 5-year survival rate for tumor thickness ≤ 1 mm and >4 mm, without other adverse prognostic factors, is 95% and 67%, respectively [7]. Similarly, when ulceration in thick (>4 mm) melanomas is present, the 5-year survival rate decrement is estimated to be 22% [7]. However, reports from various trials have indicated that a not neglectable proportion of patients with thin melanomas develop metastases [47], while patients with thick melanomas can have a decent 5-year survival rate [48]. As a result, an attempt was made to identify further specific prognostic indicators.

Based on results from other types of cancer, melanoma vascularity has been proposed as a discrete, survival affecting, factor. Melanoma vascularity and angiogenesis have been quantified with various direct or indirect methods, such as estimation of the expression of growth factors [42] and microvessel counting through light microscopy [49] or immunochemistry [11]. Bartha and Rieger [50] proposed a theoretical model that incorporated tumor growth, vessel cooption, neovascularization, vessel collapse, and cell death elements, concluding that the microvascular ecosystem is the main condition for tumor advancement. The same model suggested that, due to intratumoral vessel instability, MVD measurements are inconsistent, thus lacking any prognostic value. According to a study by Döme et al. [17], the rate of visceral metastases was associated only with the intratumoral MVD, despite the higher vascularization rate of the peritumoral tissue. Results from this study, also, showed that subjects with a high intratumoral MVD, when compared to a respective low MVD group, had a significant decrement in 5-year survival rates. Similarly, in a retrospective multicentered study, intratumoral blood vasculature had a statistically significant correlation with distant organ metastases [18]. Moreover, Aung et al. [34] suggested the existence of a correlation between MVD and host response in melanomas with BRAF mutations that could possibly influence the therapeutic model applied.

Primary studies in the field of melanoma angiogenesis claimed the existence of a correlation between MVD and survival [25, 41, 42, 51, 52]. However, later researches from various study groups concluded that MVD measurements are not a prognostic index of cutaneous melanoma survival [33, 53]. In addition to this, a recent meta-analysis by Pastushenko et al. [15] reported that there was no significant difference between the pooled MVD of the metastatic and the nonmetastatic group. Consequently, no final conclusion could be drawn, regarding the effect of vascularity on survival of melanoma patients.

Our study showed that there was no significant effect of the MVD on the hazard ratios of OS and DFS (HR: 2.62, *p* = 0.15, and HR: 2.64, *p* = 0.1, resp.). Moreover, no significant difference between high and low MVD melanomas in terms of overall survival rates at 12, 36, and 60 months (OR: 1.45, *p* = 0.74; OR: 2.93, *p* = 0.17; OR: 4.09, *p* = 0.08, resp.) was recorded. It must be noted, though, that the above-mentioned analyses suffered from a high degree of heterogeneity. Further investigation of the heterogeneity, on the basis of primary outcome, included techniques like sensitivity analysis, subgroup analysis, and metaregression. Unlike the MVD assessment antibody, age and MVD cut-off level were not found to be possible heterogeneity intriguing factors. Moreover, the nonstandardized methodology of angiogenesis qualification could explain a part of the overall heterogeneity.

These results are in accordance with various reports from the literature [54, 55]. Guffey et al. [56] showed in a comparative study, between Clark II recurrent melanomas and nonrecurrent tumors, that vascularity does not have a prognostic value. Furthermore, Hillen et al. [14] reported that CD31/CD34 MVD of both intratumoral and peritumoral areas was not associated with the tumor stage and overall survival. In a retrospective study by Shimizu et al. [44], CD34 MVD failed to provide a statistically significant prognostic value for both OS and DFS. In addition to that, Massi et al. [45] showed that although vascular density was higher in progressed cases, that discrepancy could not be confirmed statistically.

Analysis in terms of DFS, in the present meta-analysis, highlighted a significant superiority of low vasculature tumors, at all time endpoints (OR: 4.69, *p* < 0.0001; OR: 2.18, *p* = 0.004; and OR: 7.46, *p* = 0.01, resp.). Besides the measurements at 60 months' interval, all other results displayed a minimum amount of heterogeneity, thus validating their significance.

Similar results were reported from the study group of Vlaykova et al. [12] and from Valencak et al. [13], where CD31 MVD was found to be an independent prognostic factor for OS and DFS (RR: 4.324, *p* = 0.015, and RR: 3.707, *p* = 0.009, resp.). Moreover, Kashani-Sabet et al. [57] claimed that a high degree of tumor vascularity was responsible for an increased risk of relapse and mortality rate, thus deteriorating relapse-free and overall survival. Neitzel et al. [36] reported that microvessel counts were higher in the metastatic group and that 100% of the metastatic cases had a MVD ≥ 37 instead of only 8% in the nonmetastatic group. In a comparative study by Demirkesen et al. [40] a significant difference was found in the mean CD31 MVD measurements in favor of the nonmetastatic group (12.96 ± 6.02 versus 24.09 ± 5.55). Using CD31 Chalkley score for vasculature estimation, instead of MVD, Depasquale and Thompson [35] identified angiogenesis as an independent predictor of melanoma recurrence.

The prognostic value of MVD has been studied in a variety of solid tumors, apart from malignant melanoma. These include cancers of the respiratory system, breast, genitourinary tract, gastrointestinal tract, and gynecological malignancies [58]. Despite the fact that the overall trend is that MVD assessment retains a prognostic value, several studies have questioned this assumption, leaving us with no definitive conclusion on the clinical usefulness of this approach [58].

Discrepancies in methodology that are, possibly, responsible for the diversity in clinical outcomes include the choice of endothelial marker (i.e., pan-endothelial cell markers such as CD31, CD34, vWF, or factors selective for the activated/proliferating endothelium, such as CD105), the type of the fixative used, the method of MVD assessment (i.e., Weidner's hot-spot method [11], lumen method [59, 60], Chalkley's method [61], and computerized image analysis system [62]), form of vessel quantification (highest-MVD, average MVD, and microvascular volume), the designation cut-off value for increased vascularity, the magnification size (i.e., 200x, 400x), and the field size (ranging from 0.12 mm^2^ to 1.00 mm^2^) [58, 63–65]. More specifically, in melanoma, a further mechanism, by which discrepancies between MVD assessment and survival may occur, is vasculogenic mimicry [58, 66, 67]. However, from all the above possible methodological pitfalls, the selection of the hot-spot is the one thought to entail the highest interobserver variability, since it, mainly, depends on the training and experience of the investigator [63, 68].

### 4.2. Limitations

It must be noted that, before appraising the outcomes of this study, several limitations should be considered. Heterogeneity between studies was found to be in statistically significant levels, when pooled Hazard Ratio of the overall and the disease-free survival was estimated and when comparisons between the two MVD arms, in terms of survival ratios, were made. Bias in the meta-analysis could, also, be introduced through the lack of tumor stratification and inconsistent reporting of data regarding Clark's level, Breslow thickness, and overall disease stage. Furthermore, another source of potential bias might also be the nonuniform distribution between the melanoma histotypes and differences in the patient allocation, among various chemotherapeutic treatment modules. Given the fact that all the included trials were not randomized controlled studies and the majority included a retrospective design, bias due to methodological faults in the selection, comparability, and final outcome processes could influence the pooled result. Finally, since the majority of the raw data had to be extracted and reconstructed from the provided Kaplan-Meier curves, a minimum amount of bias through this process should be anticipated, despite the fact that a technique described and implemented in the current literature was used [9, 20, 21, 69].

## 5. Conclusions

To the best of our knowledge, this meta-analysis is the first to provide a pooled estimate of OS and DFS in patients with cutaneous melanoma. Our study concluded that, in melanoma patients, tumor MVD does not influence the Hazard Ratio of OS and DFS. Moreover, high and low MVD malignancies did not differ in terms of overall survival at 12, 36, and 60 months. However, low MVD tumors demonstrated a statistically significant higher rate of disease-free survival at all three time endpoints. Based on the above-mentioned results and given several limitations, further prospective studies of higher methodological and quality level and with an adequate randomization and blinding algorithm are required, in order to clarify the effect of tumor MVD in the survival of melanoma patients.

## Figures and Tables

**Table 1:** Included studies.

PMID	Author	Type of study	Year	Country	Center	Sample (patients)	Analysis sample	Specimens	Age	Gender (male/female)	Follow-up
12115882	Döme et al.	Prospective	2002	Hungary	Single centre	45	45	45 melanomas, 4 nontumorous skin biopsy samples, 3 naevi	n/a	n/a	>5 years

17013095	Hillen et al.	Retrospective	2006	Netherlands	Single centre	58	58	58	52.69 (5.5)	18 (31%)/40 (69%)	10 years
10465583	Ilmonen et al.	Retrospective	1999	Finland	Single centre	84	84	84	54.2 (11.233)	42 (50%)/42 (50%)	4.05–4.54 years

11942572	Massi et al.	Retrospective	2002	Italy	Single centre	45	45	45	Cases: 56 (8) Controls: n/a	22 (48.9%)/23 (51.1%)	Cases: 154 (21.75) months Controls: 94 (22.25) months

26264662	Pastushenko et al.	Retrospective	2015	Spain	Multicentre	196	196	196	65.2 (12.466)	103 (52.55%)/89 (45.4%)	2.7 (2.1) years

12760367	Ribatti et al.	Prospective	2003	Italy	Single centre	25	25	25	22–84	10 (40%)/15 (60%)	4-5 years

26237765	Shimizu et al.	Retrospective	2015	Japan	Single centre	128	128	158 (128 melanomas and 30 resected melanocytic naevi)	63 (12)	65 (50.78%)/63 (49.22%)	1759 days (1,221.33)

11438469	Straume et al.	Retrospective	2001	Norway	Single centre	202	174	270 (202 VGP melanomas and 68 metastases)	n/a	n/a	76 months (32.833)

14746853	Valencak et al.	Retrospective	2004	Austria	Single centre	120	120	120	57.13 (10.33)	n/a	44.6 months (15.56)

**Table 2:** MVD assessment.

Author	MVD assessment method	Antibody	Magnification used	Spots examined	Blinded reading	Observers	Separate count for intra/peritumoral vessels	MVD cut-off
Döme et al.	Immunohistochemistry, light microscopy, transmission electron microscopy	CD-31, anti-laminin	20x, 40x	2	n/a	n/a	Yes	30 microvessels/mm^2^

Hillen et al.	Light microscopy, immunohistochemistry	CD-31, CD-34	200x	4	Yes	2	Yes	n/a

Ilmonen et al.	Light microscopy, immunohistochemistry	CD-31	n/a	n/a	n/a	n/a	No	20 microvessels/mm^2^

Massi et al.	Light microscopy, immunohistochemistry	CD-31	10x, 40x	n/a	n/a	n/a	No	Microvessel area: 389.3770/*μ*m2

Pastushenko et al.	Light microscopy, immunohistochemistry, Chalkley score	CD-34	100x, 200x, 400x	3	n/a	n/a	Yes	1.67 microvessels/mm^2^

Ribatti et al.	Light microscopy, immunohistochemistry	VIII factor	250x	4–6	Yes	2	No	10 microvessels/mm^2^

Shimizu et al.	Light microscopy, immunohistochemistry	CD-34	400x	4	Yes	>2	No	4 microvessels/mm^2^

Straume et al.	Light microscopy, immunohistochemistry	VIII factor, CD105	25x, 100x, 400x	<10	n/a	n/a	Yes	94 microvessels/mm^2^

Valencak et al.	Light microscopy, immunohistochemistry	CD-31	40x, 200x	3	Yes	2	No	32.33 microvessels/field

**Table 3:** Location and Ulceration.

Author	Location						Ulceration	
	Head and neck	Trunk	Upper extremity	Lower extremity	Palm/sole	Genitals	Yes	No
Döme et al.	n/a	n/a	n/a	n/a	n/a	n/a	n/a	n/a
Hillen et al.	6 (10.3%)	17 (29.3%)	22 (37.9%)	0 (0%)	0 (0%)	0 (0%)	12 (20.6%)	46 (79.4%)
Ilmonen et al.	10 (11.9%)	35 (41.667%)	13 (15.48%)	20 (23.8%)	5 (5.95%)	1 (1.19%)	14 (16.667%)	70 (83.33%)
Massi et al.	6 (13.3%)	21 (46.6%)	3 (6.6%)	12 (26.6%)	0 (0%)	3 (6.6%)	31 (68.8%)	14 (31.1%)
Pastushenko et al.	40 (20.4%)	45 (22.95%)	92 (46.93%)	16 (8.16%)	0 (0%)	71 (36.22%)	125 (63.775%)	40 (20.4%)
Ribatti et al.	5 (20%)	13 (52%)	7 (28%)	0 (0%)	0 (0%)	0 (0%)	n/a	n/a
Shimizu et al.	0 (0%)	35 (27.35%)	93 (72.65%)			0 (0%)	21 (16.4%)	107 (83.6%)
Straume et al.	47 (25.2%)	49 (26.3%)	38 (20.4%)	52 (27.9%)	n/a	n/a	79 (42.9%)	105 (57.1%)
Valencak et al.	n/a	n/a	n/a	n/a	n/a	n/a	n/a	n/a

**Table 4:** Clark's level and Breslow thickness.

Author	Clark's level						Breslow thickness				
	0	1	2	3	4	5	1	2	3	4	5
Döme et al.	n/a	n/a	n/a	n/a	n/a	n/a	13 (28.9%), <1.5 mm		32 (71.11%), >1.5 mm		

Hillen et al.	n/a	n/a	n/a	n/a	n/a	n/a	14 (24.1%)	23 (39.7%)	12 (20.7%) 1.51–3 mm		9 (15.5%)

Ilmonen et al.	1 (1.2%)		3 (3.57%)	51 (60.71%)	25 (29.76%)	4 (4.76%)	24 (28.57%)	25 (29.76%)	34 (40,47%), >1.5 mm		

Massi et al.	0 (0%)	0 (0%)	0 (0%)	4 (8.8%)	40 (88.8%)	1 (2.2%)	Cases: 4.3 (1.23) mm Controls: n/a	0 (0%)	0 (0%)	0 (0%)	4 (8.8%)

Pastushenko et al.	n/a	n/a	n/a	n/a	n/a	n/a	58 (30.05%) ≤ 1 mm, 39 (19.89%) 1-2 mm, 44 (22.44%) 2–4 mm, 57 (29.08%) > 4 mm				

Ribatti et al.	n/a	n/a	n/a	n/a	n/a	n/a	0 (0%), ≤1.5 m		25 (100%), >1.5 mm		
Shimizu et al.	n/a	n/a	n/a	n/a	n/a	n/a	n/a	n/a	n/a	n/a	n/a

Straume et al.	n/a	n/a	n/a	84	97	100	n/a	n/a	n/a	n/a	n/a

Valencak et al.	6 (5%)	1 (0.833%)	14 (11.667%)	45 (37.5%)	51 (42.5%)	3 (2.5%)	n/a	n/a	n/a	n/a	n/a

**Table 5:** Histotype and treatment.

Author	Histotype					Treatment			
	SSM	NM	LM	ALM	Unclassified	Surgery	Surgery type	Chemotherapy	Chemotherapy type
Döme et al.	n/a	n/a	n/a	n/a	n/a	n/a	n/a	n/a	n/a

Hillen et al.	34 (58.6%)	24 (41.3%)	0 (0%)	0 (0%)	0 (0%)	Yes	Surgical excision	n/a	n/a

Ilmonen et al.	n/a	n/a	n/a	n/a	n/a	n/a	n/a	n/a	n/a

Massi et al.	27 (60%)	15 (33.3%)	0 (0%)	3 (6.6%)	0 (0%)	Yes	Surgical excision	n/a	n/a

Pastushenko et al.	n/a	n/a	n/a	n/a	n/a	Yes	Surgical excision	n/a	n/a

Ribatti et al.	n/a	n/a	n/a	n/a	n/a	Yes	Surgical excision	n/a	n/a
Shimizu et al.	n/a	n/a	n/a	n/a	n/a	Yes	Surgical excision	n/a	n/a

Straume et al.	n/a	n/a	n/a	n/a	n/a	Yes	Surgical excision	n/a	n/a

Valencak et al.	n/a	n/a	n/a	n/a	n/a	Yes	Surgical excision	No	No

**Table 6:** Newcastle-Ottawa Scale.

Study	Selection				Comparability	Exposure/outcome			Total
	1	2	3	4	5	6	7	8	
Döme et al.		*∗*	*∗*			*∗*	*∗*	*∗*	5
Hillen et al.		*∗*	*∗*			*∗*	*∗*	*∗*	5
Ilmonen et al.		*∗*	*∗*			*∗*	*∗*	*∗*	5
Massi et al.		*∗*			*∗∗*	*∗*	*∗*		5
Pastushenko et al.		*∗*	*∗*			*∗*	*∗*	*∗*	5
Ribatti et al.		*∗*	*∗*		*∗*	*∗*	*∗*	*∗*	6
Shimizu et al.	*∗*	*∗*		*∗*		*∗*	*∗*		5
Straume et al.		*∗*	*∗*			*∗*	*∗*	*∗*	5
Valencak et al.		*∗*	*∗*			*∗*	*∗*	*∗*	5

## Appendix

See Figures 1–10 and Tables 1–6.
